# Supplementary material for: Genetic Diversity and Population Structure of Rhodeus uyekii in the Republic of Korea Revealed by Microsatellite Markers from Whole Genome Assembly
Source: Int J Mol Sci. 2024 Jun 18;25(12):6689. doi: 10.3390/ijms25126689 (PMC11203500; doi:10.3390/ijms25126689)
Supplement: Supplementary file 1 [file ijms-25-06689-s001.zip › ijms-3043082-supplementary.pdf]

**Table. S1.** Collection information of 120 individuals used for population genetic analysis.

| Stream | Water system | Population ID | Number of individuals | Locations |
|---|---|---|---|---|
| Dugyecheon Stream | Geumgang River | DG | 20 | 36° 17' 14.0" N, 127° 14' 52.8" E |
| Bongseocheon Stream | Mangyeonggang River | BS | 20 | 35° 53' 20.0" N, 127° 10' 05.2" E |
| Gaecheon Stream | Yeongsangang River | GC | 20 | 35° 23' 51.9" N, 126° 48' 25.9" E |
| Geumgangcheon Stream | Tamjingang River | GG | 20 | 34° 43' 43.7" N, 126° 41' 37.1" E |
| Masancheon Stream | Somjingang River | MS | 20 | 35° 12' 07.5" N, 127° 29' 51.2" E |
| Daechocheon Stream | Yeongsangang River | DC | 20 | 34° 57' 59.9" N, 126° 51' 52.0" E |

**Table. S2.** 10 species of information used in OrthoFinder for orthologous groups analysis.

| Scientific name | GenBank WGS accession numbers |
| --- | --- |
| *Carassius auratus* | GCA_003368295.1 |
| *Carassius gibelio* | GCA_019843895.2 |
| *Cyprinus carpio* | GCA_018340385.1 |
| *Labeo rohita* | GCA_022985175.1 |
| *Onychostoma macrolepis* | GCA_012432095.1 |
| *Puntigrus tetrazona* | GCA_018831695.1 |
| *Sinocyclocheilus anshuiensis* | GCA_001515605.1 |
| *Sinocyclocheilus grahami* | GCA_001515645.1 |
| *Sinocyclocheilus rhinocerous* | GCA_001515625.1 |
| *Rhodeus uyekii* | This study |

**Table. S3.** OrthoFinder statistics of 10 species with *C. pumila*

| Statistics | |
|---|---|
| Number of species | 10 |
| Number of genes | 602,402 |
| Number of genes in orthogroups | 576,566 |
| Number of unassigned genes | 25,836 |
| Percentage of genes in orthogroups | 95.7% |
| Percentage of unassigned genes | 4.3% |
| Number of orthogroups | 31,396 |
| Number of species-specific orthogroups | 3,050 |
| Number of genes in species-specific orthogroups | 14,300 |
| Percentage of genes in species-specific orthogroups | 2.4% |
| Mean orthogroup size | 18.4 |
| Median orthogroup size | 15.0 |
| G50 (assigned genes) | 25 |
| G50 (all genes) | 24 |

| | |
|---|---|
| O50 (assigned genes) | 6,443 |
| O50 (all genes) | 6,966 |
| Number of orthogroups with all species present | 11,505 |

**Table S4.** Microsatellite information screened from *R. uyekii* genome sequence

| Result of microsatellites search | |
|---|---|
| Total number of contigs sequences examined | 2,652 |
| Total size of examined sequences (bp) | 894,559,954 |
| Total number of identified microsatellites | 842,495 |
| Number of microsatellites containing contigs sequences | 2,005 |
| Number of sequences containing more than 1 SSR | 1,954 |
| Number of SSRs present in compound formation | 437,324 |

**Table S5.** Characteristic and diversity information for the 21 microsatellite loci of *R. uyekii*

| Locus | GenBank Accession no. | Primer sequences (5'-3') | Repeat Motif | Anealing Tm (℃) | Product size | *N* | $N_A$ | $H_O$ | $H_E$ | PIC |
|---|---|---|---|---|---|---|---|---|---|---|
| RhuyMS7 | OR753515 | F: TGTAAAACGACGGCCAGTTAAGCCAAAGCCGTTTCCCT<br>R: GCAAGTGAGAGCTGGTTTG | AC(16) | 58 | 250 | 24 | 6 | 0.250 | 0.569 | 0.500 |
| RhuyMS11 | OR753516 | F: TGTAAAACGACGGCCAGTTGCTGGTCTGGTCTTGACT<br>R: TGTTTCCGTTTTGCTGCCT | GT(16) | 58 | 355 | 24 | 12 | 0.762 | 0.897 | 0.863 |
| RhuyMS4 | OR753517 | F: TGTAAAACGACGGCCAGTGGAGTCCAACTTCTCGCACA<br>R: CACACAGTTCCCCCAGAGAC | GT(18) | 58 | 196 | 24 | 15 | 0.625 | 0.895 | 0.865 |
| RhuyMS10 | OR753518 | F: TGTAAAACGACGGCCAGTGTGACGCAGATGTGAATGGC<br>R: TGGTTCCAGTCTCGCGAAAA | TC(16) | 58 | 261 | 24 | 6 | 0.409 | 0.720 | 0.663 |
| RhuyMS27 | OR753519 | F: TGTAAAACGACGGCCAGTGCCTATGGGGAATGAGCGAA<br>R: GTGGGAACGTGGGATCTGTT | AG(15) | 58 | 176 | 24 | 9 | 0.444 | 0.851 | 0.807 |
| RhuyMS15 | OR753520 | F: TGTAAAACGACGGCCAGTTGGCAGGTTGTCCAACAAGA<br>R: AACACCTGGAGTCTGTCTGC | TC(15) | 58 | 168 | 24 | 14 | 0.632 | 0.900 | 0.866 |
| RhuyMS5 | OR753521 | F: TGTAAAACGACGGCCAGTTGAACATGCCGGACCACACT<br>R: GCACTCACCTGAGCTCATGA | CA(17) | 58 | 229 | 24 | 20 | 0.750 | 0.939 | 0.914 |
| RhuyMS26 | OR753522 | F: TGTAAAACGACGGCCAGTGTCCTGCTTGCCCAAGTTTG<br>R: AGCCTGTTCCTATGGGGTCT | TC(15) | 58 | 235 | 24 | 18 | 0.667 | 0.933 | 0.907 |

| | | | | | | | | | | |
|---|---|---|---|---|---|---|---|---|---|---|
| RhuyMS28 | OR753523 | F: TGTAAAACGACGGCCAGTACACTCACGTTTAGGCCGTT<br>R: CGTCACGCACACATCATTCC | TC(15) | 58 | 244 | 24 | 11 | 0.857 | 0.892 | 0.858 |
| RhuyMS39 | OR753524 | F: TGTAAAACGACGGCCAGTAATCCAAGCCTGCGTAACCA<br>R: CACTGTGCTCCATCACTCCA | AG(15) | 58 | 245 | 24 | 10 | 0.526 | 0.755 | 0.708 |
| RhuyMS19 | OR753525 | F: TGTAAAACGACGGCCAGTTCTTCCAGTACCTGCTCACC<br>R: ACCAGGAAAGGCCAAGAACA | TC(15) | 58 | 320 | 24 | 17 | 0.783 | 0.923 | 0.895 |
| RhuyMS41 | OR753526 | F: TGTAAAACGACGGCCAGTATGTGTGTCAAGGCCCAGAG<br>R: AGGCTGGAAAATGTCAAACACA | TC(15) | 58 | 156 | 24 | 6 | 0.524 | 0.751 | 0.686 |
| RhuyMS45 | OR753527 | F: TGTAAAACGACGGCCAGTAGGCGGTGTTATGCATCCAT<br>R: GACTCCAGCAAGTCGGAACT | CA(14) | 58 | 307 | 24 | 9 | 0.565 | 0.803 | 0.760 |
| RhuyMS50 | OR753528 | F: TGTAAAACGACGGCCAGTGTGAGGCGGCTGTTTGTTTT<br>R: GGATTGGACAGGGTGCAAGA | CA(14) | 58 | 261 | 24 | 6 | 0.667 | 0.774 | 0.723 |
| RhuyMS60 | OR753529 | F: TGTAAAACGACGGCCAGTACAACCTCGACACAATAACAGT<br>R: CAACCATGCCACGCTCAAAA | TGA (10) | 58 | 284 | 24 | 15 | 0.733 | 0.926 | 0.888 |
| RhuyMS62 | OR753530 | F: TGTAAAACGACGGCCAGTGGCAGAACACACACGCTAAC<br>R: GTCAGCGACCCAATGAGGAT | TCA (10) | 58 | 315 | 24 | 5 | 0.200 | 0.574 | 0.487 |
| RhuyMS67 | OR753531 | F: TGTAAAACGACGGCCAGTGCCCACCGCCAATAAACATC<br>R: TTCTGCTGAACTACGGTGCA | AGA (10) | 58 | 256 | 24 | 8 | 0.429 | 0.788 | 0.737 |
| RhuyMS88 | OR753532 | F: TGTAAAACGACGGCCAGTTCACTCGCTTCTGCCACATT<br>R: CTGAACTTCTGACCCACGCT | AGAT(8) | 58 | 239 | 24 | 18 | 0.619 | 0.925 | 0.895 |

| | | | | | | | | | | |
|---|---|---|---|---|---|---|---|---|---|---|
| RhuyMS86 | OR753533 | F: TGTAAAACGACGGCCAGTCCCCTGCCCAATTCAGATGA<br>R: TGCTTGAGGGGAAAAGCCAT | AGGA(8) | 58 | 372 | 24 | 8 | 0.650 | 0.812 | 0.763 |
| RhuyMS89 | OR753534 | F: TGTAAAACGACGGCCAGTGCCTGGACTCACAAGACCAA<br>R: ACACCAAGTGAGCCAGTCTG | ATA (21) | 58 | 267 | 24 | 23 | 0.739 | 0.955 | 0.930 |
| RhuyMS87 | OR753535 | F: TGTAAAACGACGGCCAGTTGGGTAGGTTTAGGGGTCAGT<br>R: AGAAGTCTGCAGCCAAGACC | ATA (20) | 58 | 341 | 24 | 15 | 0.389 | 0.938 | 0.906 |

*N*: number of samples, $N_A$: number of alleles, $H_O$: observed, $H_E$: Expected heterozygosity, PIC: polymorphism information content.
